# The development of an arm activity survey for breast cancer survivors using the Protection Motivation Theory

**DOI:** 10.1186/1471-2407-7-75

**Published:** 2007-05-08

**Authors:** Teresa S Lee, Sharon L Kilbreath, Gerard Sullivan, Kathryn M Refshauge, Jane M Beith

**Affiliations:** 1 School of Physiotherapy, University of Sydney, PO Box 170, Lidcombe 1825, Australia; 2 Faculty of Education and Social Work, University of Sydney 2006, Australia; 3 Sydney Cancer Centre, Royal Prince Alfred Hospital, Missenden Rd, Camperdown 2050, Australia

## Abstract

**Background:**

Current research evidence indicates that women should return to normal use of their arm after breast cancer surgery. However, it appears some women continue to hold the view that they are supposed to protect their arm from strenuous activities because of the risk of lymphoedema. Many factors contribute to women's perceptions about lymphoedema and their ability to use their affected arm, and it is the aim of this study to explore and understand these perceptions.

**Methods/design:**

A survey, based on the Protection Motivation Theory, has been developed and tested. The survey assesses whether subjective norms, fear and/or coping attributes predict women's intention to use their affected arm. In addition, the survey includes questions regarding cancer treatment and demographic characteristics, arm and chest symptoms, and arm function. Recruitment of 170 breast cancer survivors has begun at 3 cancer treatment sites in Sydney, Australia.

**Discussion:**

This study will identify perceptions that help predict the extent women use their affected arm. The results will also determine whether upper limb impairments arise secondary to over-protection of the affected arm. Identification of factors that limit arm use will enable appropriate prevention and better provision of treatment to improve upper limb outcomes.

## Background

Breast cancer is the most common cancer affecting women aged 35 years and older, with one in eleven women expected to develop breast cancer by the age of 75 years [1]. Diagnostic and treatment advances over the past two decades have resulted in better outcomes and survival rates, however, arm pain, shoulder stiffness, and arm swelling have been reported in more than one third of women surveyed at six months or more after diagnosis [2,3]. At approximately three years following diagnosis, these impairments continue to persist, compromising activities of daily living such as the ability to do up a back-fastening bra, carry a weight, do the ironing, close a car boot, open a tight jar, and putting washing on the line [4]. These impairments are emotionally distressing because they result in an inability to resume activities of daily living.

Historically, women were advised to protect their arm to prevent development of lymphoedema [5]. Since the late 1990s, the use of minimally invasive surgery including sentinel node biopsy and the phasing out of radiotherapy to the axilla has improved upper limb outcomes substantially, such that women who are treated for breast cancer now appear to be at a much lower risk of lymphoedema [6-8]. Advice based on current research encourage women to use their affected arm normally in the early months following their surgery. In fact, more strenuous forms of arm exercises have been advised to improve upper limb strength [9-12]. Previous advice on the avoidance of strenuous arm work is no longer supported because data shows it does not exacerbate lymphoedema [9-12].

There has been some suggestion that despite the current information on lymphoedema, women do not necessarily intend to use their arm normally after surgery [13,14]. This is consistent with previous behavioural research that indicates information provided about best practice does not necessarily translate to corresponding behaviour [15-17]. Reasons for women's intention to protect their arm may include fear of lymphoedema, the information available about lymphoedema, influences or perceived social pressure from family and friends, and their ability to carry out such protective behaviour. These factors are important in understanding the extent to which women use their affected arm after breast cancer treatment. One approach to understanding these factors is to explore the women's beliefs and perceptions using a social cognition model. We have selected the Protection Motivation Theory for this study.

### The Protection Motivation Theory

**Figure 1:**
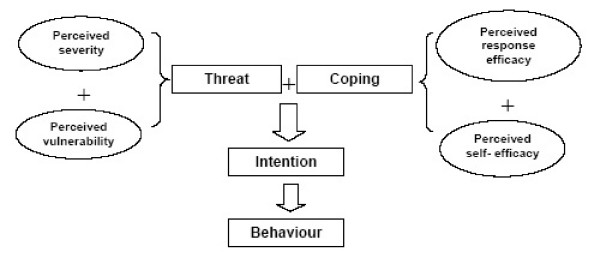
Flow chart of Protection Motivation Theory.

The Protection Motivation Theory [18] can be used to explain behaviour in relation to threat and coping relevant to health risks and behavioural intention (Figure 1). Threat refers to the extent to which people perceive they are susceptible to the health risk and their perception of the severity of the health risk. Coping refers to the extent people feel that a particular behaviour will protect them from the health risk and whether or not they feel they are able to perform such behaviour. A meta-analysis of 65 studies that used at least one component of the Protection Motivation Theory in examining health behaviours supported the structure of the model in predicting intention and behaviour [19].

In studies of compliance with medical advice, stronger and more consistent relationships were found between coping and intention than threat and intention to perform protective behaviours [19,20]. Self-efficacy had the most consistent association with intention, such that individuals were more likely to take action if they thought they could effectively perform an adaptive behaviour.

The Protection Motivation Theory is useful for understanding how perceptions contribute to intention and behaviour. The theory may be used to suggest effective educational communications to change current post-operative advice. In addition to conventional protection motivation theory, it was necessary to add a component known as "subjective norm" to this study because women may hold different interpretations of the "norms" that were established by health professionals regarding arm use following breast cancer surgery [13]. This component was derived from the Theory of Planned Behaviour [21] and it aims to assess women's perceptions about whether significant individuals such as family members, friends or health professionals think the individual should engage in a particular behaviour.

We hypothesise that upper limb impairments may arise secondary to women over-protecting their affected arm. For example, women who have high fear, i.e. view lymphoedema as a severe condition and who feel that they are vulnerable to lymphoedema, will be more likely to protect their arm, use their arm less and avoid strenuous arm activities. Conversely, women who have low fear, i.e. view lymphoedema as less severe and who feel they are not vulnerable to lymphoedema, will be more likely to engage in strenuous arm activities and will be less likely to engage in protective behaviours for their arm. To test these hypotheses, a survey described below was developed.

## Methods/Design

The aim of this study is to identify the perceptions of breast cancer survivors toward arm activity and strenuous arm exercise at 6–15 months after surgery. This time interval reflected the period when women were most likely to return to normal life including their work status following breast cancer treatment. The concepts of perceived threat, coping, norms and intention were operationalised to measurable statements according to Table 1. For example, the concept of perceived threat of lymphoedema was divided into perceived severity of and vulnerability to lymphoedema. Perceived severity of lymphoedema was operationalised into statements assessing the appearance of lymphoedema and effects on lifestyle. Perceived vulnerability to lymphoedema was operationalised into statements assessing the likelihood of lymphoedema affecting the participant or others undergoing the same treatment as the participant.

Some statements in the survey were based on previous studies that have used Protection Motivation Theory, and were adapted to ensure consistency with the aims of our study [22]. Other statements were newly created and were based on our clinical experience with breast cancer survivors. Commonly voiced opinions were converted into the following statements: "I will be happy to live with limited shoulder range as a result of breast cancer treatment" and "Arm weakness or restriction does not worry me as much as arm swelling does". Strenuous arm exercise was defined as exercising with a 2 kg arm weight (eg. large milk container) 20 times above head level or stretching until a pull could be felt and holding it for 30 seconds. These examples involved concepts (weights, repetition, feeling of pull) in which there was patient concern about lymphoedema risk in previous studies [10,13].

Participants are asked to indicate their belief about each statement by circling one response on a five-point Likert scale; strongly agree, agree, neither agree or disagree, disagree, and strongly disagree. This scale is advocated by social researchers [23,24], and has been used in several studies employing the Protection Motivation Theory [25-27]. An advantage of this format is that the response categories provide ordinal data. The relative strength of agreement intended by the various respondents can be judged, and the intensity of agreement between statements compared. Other studies have used Likert scales but changed the response adjectives for different statements, e.g. Completely unimportant/extremely important, completely unconfident/extremely confident [22,25,28]. Although changes of the response adjectives allow for flexibility in the development of statements, it may be confusing to participants who have to adapt to different scales across a number of statements. In this study, statements were devised to fit the agree/disagree scale to avoid confusion.

### Reliability and validity

To increase the validity and reliability of the survey instrument, 10 participants tested the survey in accordance with recommended procedures prior to sample distribution [23,24,29]. The aim of the survey pre-test was to maximise face validity by ensuring the statements were uni-dimensional and correctly interpreted. The pre-test process was divided into three phases involving test, revision and re-test. Three women were observed and the survey completion timed without any interruption from the researcher. Four women were asked to complete the survey whilst "thinking aloud". The researcher quietly observed and noted any hesitation, confusion, skipped questions, or sequencing problems associated with completing the survey. Following survey completion, participants were interviewed to assess their interpretation of questions, in particular, the questions associated with hesitation. They were also asked to report any problems they had with the content or format of the survey.

As a result of the initial pre-test, any questions that appeared ambiguous were modified to maximise participant understanding and to ensure validity. Multiple statements were added to measure each component of the theory as internal consistency between multiple item indicators is regarded as the best and most efficient method of increasing reliability in questionnaires [23,24]. Lay terms were chosen to replace more technical terms, such as "Radiation Doctor" instead of "Radiation Oncologist" and "removal of entire breast" instead of "mastectomy". Responses such as the "Encore exercise group" and "Internet" were added as categories in questions asking about sources of advice and exercise. Questions which asked "What is your age?" were replaced with "What is your age bracket?" to encourage accurate reporting and to avoid missing data. As some participants had left the last few questions incomplete, the more crucial sections such as treatment variables, perceptions and symptoms were moved to the front of the survey, while less crucial information about patient demographic variables was moved to the final section of the survey. Questions regarding occupation and educational level were added to the survey to allow for subgroup analyses. After several changes, the revised survey was re-tested on three different women successfully. At this time, the research team reached a consensus that the participants appropriately understood the statements and that the survey was ready for distribution.

### Participants

One hundred and seventy participants will be recruited at their medical follow-up appointment at three hospitals. Participants will be included if they had surgery for breast cancer 6–15 months previously, have had no recurrences since breast cancer surgery, are female, and can read and comprehend English. Women who have had bilateral breast cancers surgeries will be asked to complete the survey with reference to the side of most recent surgery. Ethical approval was obtained from Sydney South West and Northern Sydney Central Coast Area Health Service.

Suitable participants will be identified by their medical specialist and asked to complete the survey in the waiting room. No identifying information will be required or recorded for the survey. A researcher will be present in the waiting room and be available for help if required by the participant. For participants who cannot finish the survey in the waiting room, a reply-paid envelope will be provided. The survey consists of 45 questions with an estimated completion time of 30 minutes.

### Statistical Analysis

The primary endpoint of the survey is to describe women's intention to protect their affected arm after surgery for breast cancer. Women's intention to be cautious with their affected arm and their intention to avoid strenuous activities with their affected arm will also be examined. A sample size of 170 is chosen to ensure that power is sufficient to detect a 25% (95% CI 0.1–0.4) difference in women's intention to protect their arm when comparing respondents who are fearful and who are not fearful of developing lymphoedema, allowing for 10% incomplete surveys. It is assumed that there will be approximately equal numbers of respondents who are fearful and who are not fearful of developing lymphoedema.

Factor analysis will be used to identify characteristic beliefs about the lymphoedema and musculoskeletal problems from the perception statements. The minimum eigenvalue will be set at 1 and appropriate rotations will be performed to minimise the correlation among factors. Minimum factor loadings of 0.5 will be required for items to be included in a factor. Only scales with reliability measured by Cronbach's alpha of >0.6 will be used in further analysis. Descriptive statistics (mean ± standard error) will be presented for each of the common factors. Independent sample t-tests or chi-square tests will be used to determine if there is a difference in treatment, demographic variables, source of advice, mean factor scores, symptoms and function between women who intend to protect their arm and women who do not. Two-tailed significance level will be set at p > 0.05. Logistic regression will be performed to examine predictors of intention to protect arm.

## Discussion

Research evidence indicates upper limb exercise is beneficial after breast cancer surgery, improving shoulder range of motion, arm strength and quality of life [9-12]. Prolonged inactivity of the upper limb can cause short-term impairments following surgery to extend indefinitely and worsen, compounding the physical and psychological distress for breast cancer survivors. The inability to resume normal life due to physical restriction is frustrating and distressing and has been strongly associated with poorer quality of life [30].

There are no prospective studies that substantiate an adverse relationship between physical activity and lymphoedema [31], yet some women continue to protect their affected arm by avoiding strenuous types of physical activity [13,14]. The reasons behind such behaviour is not clear and may be due to a variety of perceptions women hold about lymphoedema, upper limb activity and exercise. The Protection Motivation Theory provides a valuable framework to enhance our understanding of the reasons behind women's intention to protect or use their affected arm after breast cancer treatment. The aim of this survey is to identify the factors that positively and negatively influence breast cancer survivors' perceptions toward arm activity and to provide information to enable better provision of services and treatment to reduce upper limb problems.

## Competing interests

The authors declare that they have no competing interests.

## Authors' contributions

TSL conceived and drafted the questionnaire. She is responsible for the recruitment of patients, data entry and analysis. SLK, GS, KMR and JMB contributed to the study design, refining the survey, its analysis, and manuscript development. GS provided his expertise on theory testing, survey research, and survey analysis. JMB advised on the practicalities of administering the survey at the research sites and is involved in the recruitment of patients. All authors have read and approved the manuscript.

## Pre-publication history

The pre-publication history for this paper can be accessed here:



## Figures and Tables

**Table 1:** Operationalisation of survey statements

**Theory construct**	**Appraised by**	**Survey statements**
Perceived Threat of lymphoedema	Perceived severity of lymphoedema	■ Having arm swelling would or does significantly alter my lifestyle ■ Arm swelling is a minor side-effect ■ People with arm swelling suffer a lot ■ Swelling in the arm can look very severe ■ The possibility of developing or worsening arm swelling worries me
	
	Perceived vulnerability to lymphoedema	■ I am not at risk of developing or worsening arm swelling ■ My chances of developing or worsening arm swelling is low ■ People who have had the same breast cancer treatment as me are vulnerable to arm swelling ■ It is unlikely that swelling will occur or worsen in my arm

Perceived coping by the response of protecting arm	Perceived response efficacy	■ Arm swelling is inevitable and there is little anyone can do to prevent it ■ Doing strenuous activities with my affected arm puts me at risk of developing or worsening arm swelling. ■ Repetitive arm activities will not cause arm swelling ■ Resting my affected arm will increase my chances of developing or worsening arm swelling
	
	Perceived self-efficacy	■ I am confident in my ability to avoid strenuous work ■ Strenuous work is part of my lifestyle so I must do it regardless of any advice given ■ I will rest and protect my affected arm even though it may be inconvenient

Perceived threat of musculoskeletal symptoms	Perceived severity of musculoskeletal symptoms	■ My quality of life will be reduced if arm weakness or restriction developed following breast cancer treatment ■ I will be happy to live with limited shoulder range as a result of breast cancer treatment ■ Arm swelling or restriction does not worry me as much as arm swelling does.
	
	Perceived vulnerability to musculoskeletal symptoms	■ Other people who had the same surgery as me have problems with shoulder restriction ■ I may get arm weakness following breast cancer treatment ■ Stiff shoulders are not common after breast cancer treatment

Perceived coping by the response of performing strenuous arm exercise	Perceived response efficacy	■ Doing strenuous arm exercises will help me gain arm strength and prevent weakness ■ In my opinion, you must exercise the arm strenuously to achieve full shoulder range
	
	Perceived self-efficacy	■ I can quite easily perform a strenuous exercise routine to strengthen and stretch my arm muscles ■ I am confident in doing a strenuous exercise routine by myself

Perceived norms of arm protection versus strenuous arm exercises	Perceived norms of arm protection versus strenuous arm exercises	■ Health professionals have told me to be protective of my arm and avoid strenuous arm work ■ My family and friends have told me to rest my arm ■ Health professionals approve of strenuous arm exercises

Intention	The intention to engage in arm protective behaviours	■ I plan to avoid any strenuous arm activities ■ I plan to protect my arm in any way possible so as not to develop or worsen arm swelling ■ I use my arm more cautiously now compared to before my breast cancer interventions
	
	The intention to engage in strenuous arm exercises	■ I plan to do strenuous arm exercise ■ I plan to do lighter, easier arm exercises ■ It is important for me to return to using my arm the same way as I would have before breast cancer treatment
